# Heterogeneity of cancer-associated fibroblasts and roles in the progression, prognosis, and therapy of hepatocellular carcinoma

**DOI:** 10.1186/s13045-019-0782-x

**Published:** 2019-09-23

**Authors:** Zeli Yin, Chengyong Dong, Keqiu Jiang, Zhe Xu, Rui Li, Kun Guo, Shujuan Shao, Liming Wang

**Affiliations:** 1 Engineering Research Center for New Materials and Precision Treatment Technology of Malignant Tumors Therapy, Dalian Medical University, Dalian, 116027 Liaoning China; 2 Engineering Technology Research Center for Translational Medicine, Dalian Medical University, Dalian, 116027 Liaoning China; 3 Division of Hepatobiliary and Pancreatic Surgery, Department of General Surgery, The Second Affiliated Hospital of Dalian Medical University, 467 Zhongshan Road, Dalian, 116027 Liaoning China; 4 Department of Pathology, The Second Affiliated Hospital of Dalian Medical University, 467 Zhongshan Road, Dalian, 116027 Liaoning China; 5 Key Laboratory of Proteomics, Dalian Medical University, 9 West Lvshun South Road, Lvshunkou District, Dalian, 116027 Liaoning China

**Keywords:** Hepatocellular carcinoma (HCC), Tumor microenvironment (TME), Cancer-associated fibroblasts (CAFs)

## Abstract

Hepatocellular carcinoma (HCC) is a lethal disease, and recurrence and metastasis are the major causes of death in HCC patients. Cancer-associated fibroblasts (CAFs), a major stromal cell type in the HCC microenvironment, promote HCC progression, and have gradually become a hot research topic in HCC-targeted therapy. This review comprehensively describes and discusses the heterogeneous tissue distribution, cellular origin, phenotype, and biological functions of HCC-associated fibroblasts. Furthermore, the possible use of CAFs for predicting HCC prognosis and in targeted therapeutic strategies is discussed, highlighting the critical roles of CAFs in HCC progression, diagnosis, and therapy.

## Background

Primary liver cancer is the fourth leading cause of cancer death worldwide, with a 5-year survival rate less than 20% in most countries that has changed very little over the past years [1, 2]. Hepatocellular carcinoma (HCC) comprises 75–85% of primary liver cancers and is thus the most common histological type observed in clinical practice [1]. As HCC progression has become the main cause of the poor prognosis of HCC patients [3], clarifying the factors that influence HCC progression and exploring appropriate interventions and therapies will improve the prognosis of HCC patients.

Solid tumor tissue consists of heterogeneous tumor cells and stroma. The tumor stroma is composed of blood and lymphatic vessels, nerves, the extracellular matrix (ECM), other non-cellular elements and stromal cells, providing a suitable environment for the survival of tumor cells that is termed the tumor microenvironment (TME). Tumor progression is not a fully autonomous process, and complex interactions between tumor cells and stromal components, especially stromal cells in the TME, greatly affect tumor progression; these interactions have gradually become a hot research topic in tumor-targeted therapy [4, 5]. Stromal cells in the HCC microenvironment are mainly divided into three subclasses: cancer-associated fibroblasts (CAFs), angiogenic cells, and inflammatory and immune cells. Crosstalk between these cell types and HCC cells substantially promotes tumor cell proliferation, migration, and invasion and vasculogenic mimicry (VM), inhibits tumor cell apoptosis, activates angiogenesis, and creates an immunosuppressive microenvironment, which together determines the fate of HCC [6, 7].

CAFs comprise a heterogeneous group of activated fibroblasts in the TME with different cellular origins and phenotypes that are closely associated with tumor initiation and progression. Indeed, CAFs are derived from a variety of cell types, such as endothelial cells (ECs), pericytes, vascular smooth muscle cells, cancer cells that undergo the epithelial-mesenchymal transformation (EMT), tissue residual fibroblasts, and bone marrow-derived cells. Although CAFs lack specific cell surface markers, most express α-smooth muscle actin (α-SMA), fibroblast activation protein (FAP), vimentin, and fibroblast-specific protein 1 (FSP-1) [8, 9]. CAFs are the major stromal cell type in the TME and the main source of collagen-producing cells, which not only provide mechanical support but also influence tumor cell proliferation, apoptosis, migration, invasion, angiogenesis, immune escape, and drug resistance through communication with tumor cells or other stromal cell types. Because of these characteristics, CAFs are the main stromal cells that affect tumor progression and are therefore an ideal target for tumor-targeted therapy [8]. CAFs in pancreatic ductal adenocarcinoma promote tumor progression by secreting ECM components to reshape the tumor stromal microenvironment to protect tumor cells from chemotherapy and radiation-induced damage and are involved in recruitment and programming of immune cells to create and sustain an immunosuppressive environment [10]. CAFs in colorectal cancer promote tumor development and progression by regulating intestinal inflammation, epithelial proliferation, stem cell maintenance, angiogenesis, extracellular matrix remodeling, and metastasis [11]. CAFs in gastrointestinal cancer not only directly confer growth advantages to cancer cells via paracrine signaling, exosome transfer, or physical interaction but also directly communicate with other stromal cells to create an immunosuppressive TME, promote angiogenesis, and enhance ECM remodeling and therapeutic resistance to promote tumor progression [12]. Moreover, CAFs in breast cancer contribute to drug resistance, reduce anti-tumor immunity, and are associated with aggressive tumor behavior and disease recurrence [13]. CAFs in non-small cell lung cancer promote EMT and chemoresistance among cancer cells and enhance stemness to promote tumor progression [14, 15]. The majority (70–90%) of HCC cases occur in a background of liver cirrhosis [16] caused by the activation, proliferation, and accumulation of fibroblasts [17]. Therefore, the HCC microenvironment is rich in stromal fibroblasts, and many studies over the past several years have documented that CAFs play critical roles in HCC progression and have increasingly focused on CAF-targeted therapy [18]. A few years ago, Kubo et al. summarized the biological function and molecular pathways of CAFs in HCC progression [19], and we extend this knowledge by discussing recent research progress. In this review, we comprehensively summarize studies on the distribution of CAFs in the HCC microenvironment, their heterogeneous cellular origin, CAF phenotypes and the effects of CAFs on HCC progression. We also highlight the value of CAFs in predicting HCC prognosis and their possible use as therapeutic targets, clarifying the location, origin, phenotype, and function of CAFs in the HCC microenvironment and their potential clinical applications in HCC diagnosis and therapy.

## The tissue distribution of CAFs in the HCC microenvironment

**Fig. 1:**
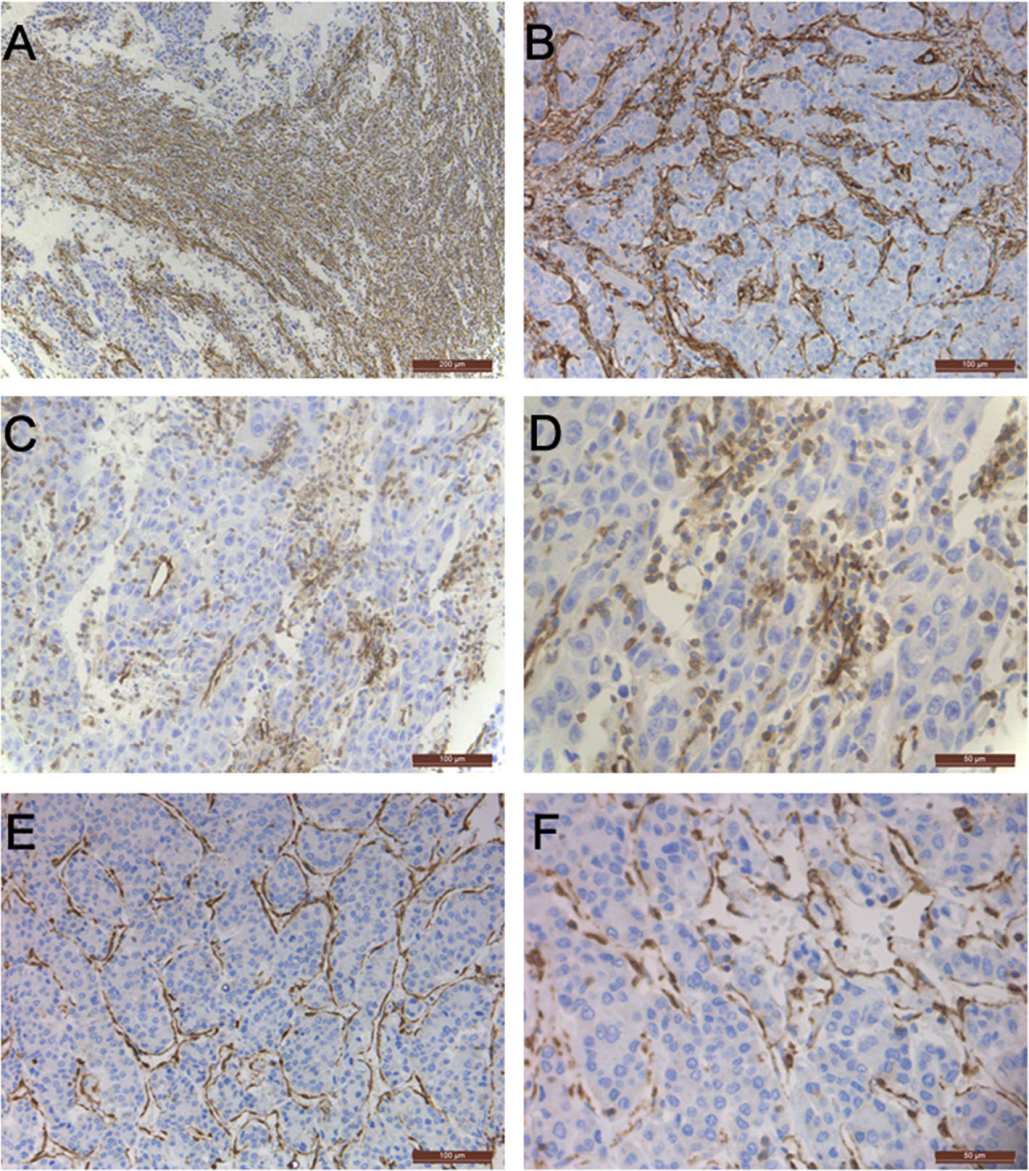
The distribution of α-SMA-positive CAFs in HCC tissues. **a** CAFs located in the tumor fibrous capsule (× 100). **b** CAFs located in the tumor fibrous septum (× 200). **c**–**d** HCC cells positive for α-SMA expression (× 200, × 400, respectively). **e**–**f** CAFs in the blood sinus (× 200, × 400, respectively)

Although CAFs are one of the major stromal cell types in the HCC microenvironment [20, 21], the exact location of CAFs in HCC tissues, which reflects and may directly determine the heterogeneous cellular origin and functions of CAFs, has not been fully characterized and has not attracted sufficient attention. The location of CAFs in the TME is often detected by immunohistochemistry, which includes several different visualization methods, such as enzyme-linked immunohistochemical staining and immunofluorescence (IF) histochemical staining, and involves probing for detection of common protein markers in CAFs, such as α-SMA and FAP [22]. Until now, most studies determining the distribution of CAFs in HCC tissues by immunohistochemistry have simply indicated their distribution in the tumor fibrous stroma without determining their precise location, instead focusing on the numbers of CAFs and their relationships with HCC clinicopathological features and prognosis [23–25]. In our previous study, we performed immunohistochemical staining of 57 HCC tissues and confirmed that α-SMA-positive CAFs in the HCC microenvironment were mainly distributed in the HCC fibrous septum, fibrous capsule, and hepatic blood sinusoids; we also observed the presence of α-SMA-positive HCC cells near the blood sinusoid in some HCC tissues (data not published) (Fig. 1). Lin et al. and Fang et al. also detected α-SMA-positive cells in the cancer cell nest, hepatic blood sinus, and sporadic HCC cells [25, 26]. However, because CAFs lack specific protein markers, we can only observe the exact tissue distribution of some CAFs by immunohistochemistry; therefore, searching for more specific markers will be beneficial for improving our knowledge of the location of CAFs in the TME and increasing their accessibility. Identifying and studying CAFs in different locations in HCC tissues, regardless of their different cellular origins, discrepant cell phenotypes, and different multidirectional functions in HCC progression, may allow us to further recognize their heterogeneity.

## Heterogeneous cellular origins of CAFs

**Fig. 2:**
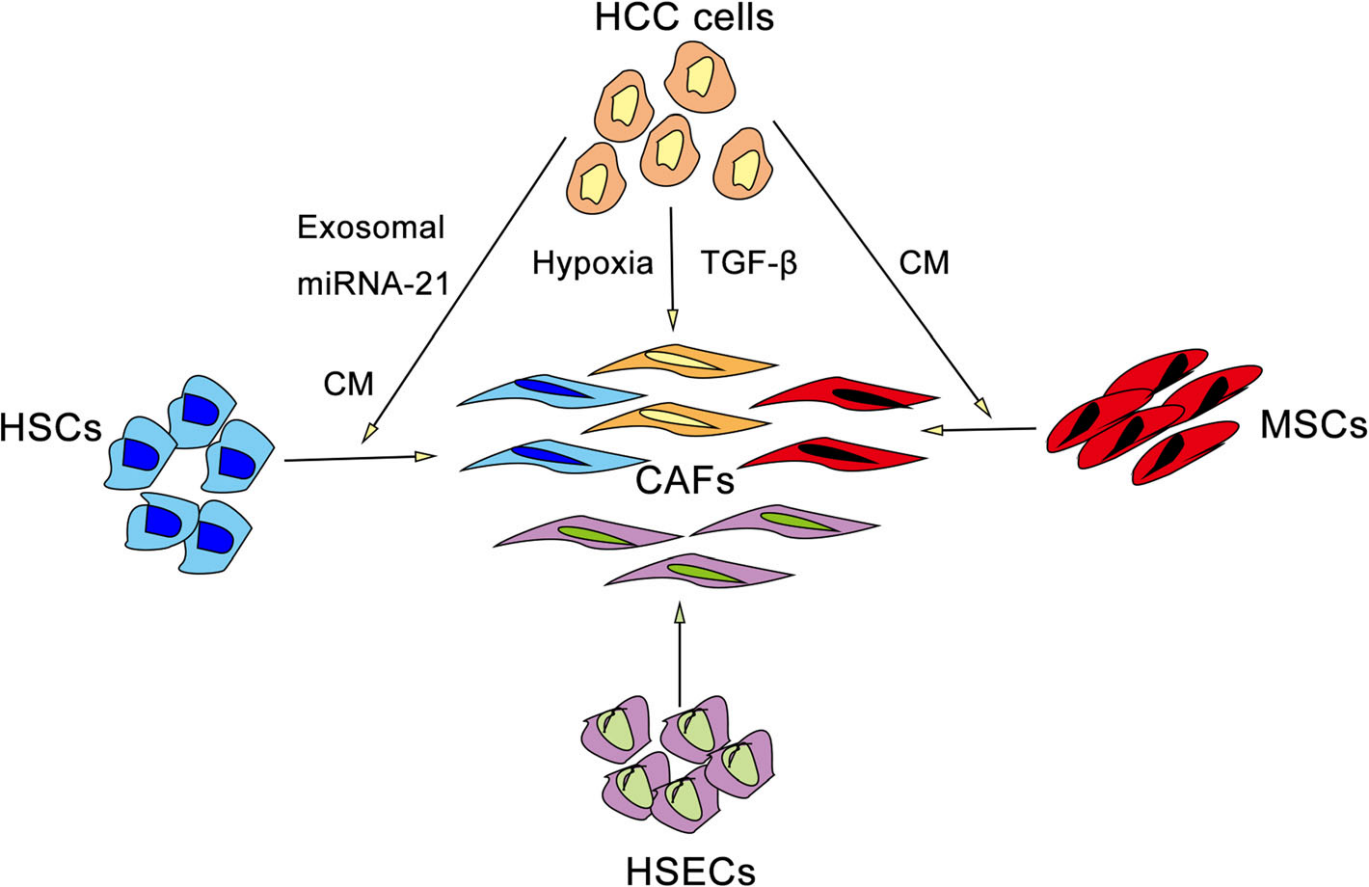
Heterogeneous cellular origins of CAFs. CAFs in the HCC microenvironment may be derived from HCC cells, HSCs, MSCs, and HSECs. Hypoxia and TGF-β can induce EMT and expression of α-SMA and FAP in HCC cells. HSCs can differentiate into CAFs under stimulation with CM or exosomal miRNA-21 secreted by HCC cells. CM generated from HCC cells can also induce the expression of tenascin-C and SDF-1 in MSCs. HSECs are a potential cellular origin of CAFs in the HCC microenvironment

CAFs in solid tumor tissues originate from a variety of cell types, mainly the following: tissue residual fibroblasts, such as pancreatic stellate cells and hepatic stellate cells (HSCs) [27, 28]; mesenchymal stem cells (MSCs) derived from human normal tissues, such as adipose and bone marrow [29, 30]; ECs that undergo the endothelial to mesenchymal transition (EndMT) [31]; and cancer cells that undergo EMT [32]. CAFs in the HCC microenvironment also have heterogeneous cellular origins (Fig. 2). As mentioned above, CAFs in the HCC microenvironment have different tissue distributions and are mainly distributed in the tumor fibrous septum, fibrous capsule, and hepatic blood sinusoids. α-SMA-positive HCC cells are also found near the blood sinusoid in some HCC tissues, which may reflect their heterogeneous cellular origins. α-SMA expression in some HCC cells indicates that CAFs may originate from HCC cells undergoing EMT; additionally, their location near the blood sinusoid suggests their association with the migration and invasion of HCC cells. Zou et al. reported that HCC cells under hypoxic conditions presented upregulated FAP expression and a classical EMT phenotype characterized by downregulated expression of the epithelial marker E-cad and upregulated expression of the mesenchymal markers twist and snail [33]. Moren et al. also found that TGF-β, a classic cytokine that induces EMT, can upregulate expression of α-SMA in HCC cells [34, 35]. Furthermore, abnormal activation, proliferation, accumulation, and migration of HSCs, the major fibrogenic cells during liver damage, cause hepatic fibrosis and cirrhosis [36]. Tumors are considered “wounds that never heal,” and most HCC cases are caused by chronic liver diseases with varying degrees of chronic inflammatory fibrosis [16, 37]. This characteristic suggests that CAFs derived from activated HSCs may be one of the main fibrogenic cell types in the HCC microenvironment and that the generation of HCC fibrous septa and fibrous capsules may be caused by CAFs derived from activated HSCs. For example, Zou et al. reported that conditioned medium (CM) generated from hepatoma cells (Huh7 cells) induced transition of the LX2 human HSC cell line into CAFs, which was confirmed by upregulated expression of α-SMA and FAP [38]. According to Zhou et al., HCC cells release exosomes into CM that induce microRNA21-mediated differentiation of LX2 cells into CAFs; in addition, differentiated CAFs exhibit high levels of proliferation and migration and increased contractile activity, secrete more proinflammatory cytokines, and promote HCC growth in subcutaneously transplanted tumors [39]. MSCs migrate to areas of liver fibrosis and the HCC microenvironment, where they participate in HCC initiation and progression [7]. After co-culture with HCC cells, MSCs present a CAF phenotype and exhibit upregulated expression of tenascin-C and SDF-1, and the co-cultured HCC cells display an EMT phenotype [40]. In addition, the distribution of CAFs in hepatic blood sinusoids in HCC tissues suggests that hepatic sinusoidal endothelial cells (HSECs) that undergo EndMT may be among the main cellular origins of CAFs in the HCC microenvironment. Although we already have a general understanding of the cellular origins of CAFs in the HCC microenvironment, their exact cellular origins remain unclear, and further clarification of the mechanisms by which cells are transformed into CAFs will be beneficial for improving our awareness of the heterogeneous cellular origins of CAFs and allowing the recognition of their discrepant phenotypes and functions.

## Phenotypic characteristics of CAFs in HCC tissues

To better distinguish the phenotype and biological function of CAFs in the HCC microenvironment, many researchers have attempted to isolate these cells from fresh HCC tumor tissues (Table 1). Primary CAF cultures have mainly been prepared by tissue culture and monolayer culture of single-cell suspensions isolated from collagenase-digested HCC tissues with or without antifibroblast micromagnetic bead sorting [41, 42, 47]. CAFs isolated from fresh HCC tissues have a spindle-shaped fibroblastic morphology and present an activated myofibroblast phenotype with expression of α-SMA, FAP, vimentin, FSP-1, platelet-derived growth factor receptors (PDGFRs), desmin, fibronectin, and collagen1α, which are common protein biomarkers of CAFs [41, 45, 46, 54, 56]. Because there are no unequivocal protein markers for CAFs, a combination of several proteins is usually detected to identify their phenotype and purity. For example, Li et al. showed that CAFs isolated from HCC tumor tissues express α-SMA, FAP, and vimentin but do not express CD31 or CD68 by IF [43]; based on by IF and flow cytometry, Lau et al. found that HCC-associated fibroblasts express α-SMA and FAP but do not express CD31, AFP, or pancytokeratin [48]. Notably, CAFs isolated from fresh HCC tumor tissues exhibit an MSC phenotype, which manifests as increased clonogenic capacity; expression of CD73, CD90, CD105, CD44, CD13, CD29, and CD166; lack of CD31, CD34, CD45, CD117, and HLA-DR expression; and multipotent differentiation, such as adipogenic, osteogenic, and pancreatic differentiation [54, 55]. However, it remains to be determined whether only a subtype of CAFs derived from MSCs or all CAFs in the HCC microenvironment are able to acquire stemness during tumor progression. Overall, the use of more advanced technologies, such as single-cell sequencing [57], to identify the phenotypic heterogeneity of HCC-associated fibroblasts may benefit CAF-targeted therapy.

**Table 1:** Phenotype and biological function of CAFs in the HCC microenvironment

Phenotype	Protein markers	Function		Molecular mechanism	Reference
Activated myofibroblast phenotype	α-SMA, collagen 1α, fibronectin	Maintain and enhance the stemness of HCC cells	In vitro: promote HCC cell proliferation, invasion, EMT In vivo: promote tumorigenesis, growth and metastasis	COMP	Sun et al. [41]
	α-SMA, vimentin		In vitro: promote proliferation, invasion	IL-6, IL-8, CCL2	Mono et al. [42]
	α-SMA, FAP, vimentin		In vitro: increased sorafenib resistance, proliferation rate, migration, and invasion In vivo: promote tumorigenesis	HGF/c-Met/STAT3 IL-6/IL6R/STAT3	Li et al. [43]
	α-SMA, FAP, vimentin		In vitro: promote proliferation, migration and invasion, and the expression of stemness genes In vivo: promote tumorigenesis and HCC growth	IL-6/STAT3/notch	Xiong et al. [44]
	α-SMA, PDGFR-α		Maintain and enhance the stemness of HCC cells	Notch 3 signaling	Liu et al. [45]
	α-SMA, FSP1, vimentin		In vivo: promote HCC initiation and growth	FOXQ1/NDRG1/CCL26 feedback loop	Luo et al. [46]
	α-SMA, FSP1, vimentin		In vitro: promote migration, invasion, EMT In vivo: promote metastasis	CCL2/CCL5/Hh, CCL7/CXCL16/TGF-β pathway	Liu et al. [47]
Activated myofibroblast phenotype	α-SMA, FAP	Maintain and enhance the stemness of HCC cells	In vitro: promote proliferation, self-renewal In vivo: promote tumorigenesis	HGF/c-Met/Erk/FRA1/HEY1 signaling	Lau et al. [48]
	α-SMA, FAP, vimentin		In vitro: promote proliferation In vivo: promote growth	HGF	Jia et al. [49]
	α-SMA, vimentin	Enhance HCC blood supply	Promote vasculogenic mimicry	TGF-β, SDF-1	Yang et al. [50]
	α-SMA, FAP, vimentin	Immunosuppression	Recruit and promote the differentiation of neutrophils, monocytes, and dendritic cells into cells with immunosuppressive phenotypes	Recruitment: SDF-1α/CXCR4 Education: IL-6/STAT3	[51–53]
Mesenchymal stromal cell phenotype	Positive: CD90, CD73, CD105, CD29, CD44, CD166 Negative: CD34,CD31, CD45, HLA-DR	Immunosuppression	Attenuate the cytotoxic activity of NK cells (downregulation of granzyme B and perforin)	PEG2, IDO	Li et al. [54]
	Positive: CD90, CD44, CD29, CD13, CD105, CD166 Negative: CD34, CD45, CD117 multipotent differentiation: adipogenic, osteogenic, and pancreatic differentiation	–	–	–	Sukowati et al. [55]

## Biological functions of CAFs in HCC progression

CAFs not only directly affect HCC cells but also communicate with other stromal cells to remodel the HCC microenvironment to influence HCC progression (Table 1). Many studies have shown that CAFs isolated from fresh HCC tumor tissues maintain and enhance the stemness of HCC cells, as manifested in their proliferation, self-renewal, migration, invasion, drug resistance, tumorigenesis, and metastasis through several different paracrine mechanisms. Sun et al. demonstrated that HCC-associated fibroblasts secrete cartilage oligomeric matrix protein (COMP) to promote proliferation, migration, invasion, and EMT in HCC cells in vitro as well as tumorigenesis, growth, and metastasis in vivo [41]. Li et al. reported that CAFs enhance the stemness of CD24^+^ HCC cells through the paracrine factors HGF and IL-6 to activate STAT3 signaling [43]. In addition, Xiong et al. and Lau et al. showed that the stemness of HCC cells was enhanced by IL-6 and HGF secreted by CAFs, possibly through the Notch and Erk signaling pathways, respectively [44, 45, 48], and Liu et al. determined that CAFs promote migration, invasion, and EMT in HCC cells in vitro and HCC cell metastasis in vivo through the CCL2/CCL5-Hh and CCL7/CXCL16-TGF-β pathways [47]. Moreover, interaction between HCC and CAFs has been shown to act as a positive feedback loop; CAFs promote the initiation and growth of HCC in vivo by inducing expression of forkhead box Q1 (FOXQ1) and therefore transactivating N-myc downstream-regulated gene 1 (NDRG1) in HCC cells, which induces CCL26 secretion and thus recruits more CAFs to promote HCC progression [46].

VM (vasculogenic mimicry) refers to the formation of a vascular-like structure by aggressive tumor cells through self-deformation and ECM remodeling that serves as a special blood supply for malignant tumors [58]. CAFs isolated from fresh HCC tissues also have the ability to induce VM in HCC cells through the paracrine factors TGF-β and SDF-1 to support the tumor blood supply [50]. Further clarification of whether CAFs participate in the formation of other blood supplies, such as those formed in angiogenesis, may be beneficial for HCC-targeted treatment.

CAFs also recruit inflammatory and immune cells, such as neutrophils, monocytes, and dendritic cells (DCs), and encourage the development of immunosuppressive phenotypes in these cells to foster HCC immune escape [51–53]. CAFs have been shown to recruit peripheral blood neutrophils, DCs, and monocytes through the SDF1α/CXCR4 pathway; activate PD-L1 expression in neutrophils; promote the acquisition of a tolerogenic phenotype in DCs; and induce the differentiation of monocytes into myeloid-derived suppressor cells (MDSCs) through the IL-6/STAT3 pathway to suppress T cell immunity [51–53].

Fibroblasts that affect HCC progression are not only restricted to CAFs in HCC tumor tissues, peritumor fibroblasts (PTFs), and fibroblasts at the site of metastasis but also have an effect on HCC progression. Zhao et al. found that PTFs promote the proliferation, migration, and invasion of HCC cells more than CAFs do, and this promotion may be mediated by increased levels of IL-6 and activation of IL-6/STAT3 signaling [59]. Jiang et al. also showed that PTFs promote in vitro migration and invasion of HCC cells and their in vivo tumorigenesis and metastasis [60]. Moreover, lung fibroblasts activated by HCC-derived exosomal miR-1247-3p acquire a phenotype similar to that of CAFs, creating a premetastatic niche suitable for lung metastasis [61].

All of the above studies suggest that CAFs enhance HCC progression, though whether all CAFs promote HCC progression or the existence of different subtypes that are inhibited in HCC progression requires further clarification. Additionally, increased knowledge of the TME in both the primary location and the site of metastasis will improve our understanding of tumor recurrence and metastasis and provide more strategies for inhibiting tumor initiation and progression.

## The value of CAFs in predicting HCC prognosis

HCC prognosis is very poor worldwide, and identifying additional sensitive, specific factors associated with HCC prognosis with the potential to predict the survival of HCC patients will benefit individualized treatment of this disease. The predictive value of the biological characteristics and behaviors of hepatoma cells in HCC, such as their degree of differentiation, microvascular invasion, and formation of hepatic stellate nodules, has attracted ample attention, and many guidelines have recognized these characteristics as pathological indicators of HCC prognosis [62–64]. Tumor stromal components provide a suitable environment for the survival of HCC cells. Regardless, it needs to be further evaluated whether these tumor stromal components are associated with HCC prognosis and can thus serve as pathological indicators to predict HCC prognosis. CAFs, one of the major stromal cell types in the HCC microenvironment, have been shown to enhance the stemness of HCC cells, promote proliferation and metastasis, and induce VM and immunosuppression, strongly promoting HCC progression. Further clarification of the relationship between CAFs and HCC prognosis and of their value in predicting the survival of HCC patients will accelerate the transformation of fundamental research on CAFs to their clinical application. Nonetheless, there have been contradictory results regarding the effect of CAFs in predicting HCC prognosis, though most studies have shown that CAFs are negatively associated with HCC survival (Table 2). For example, Lau et al. observed a significantly shorter disease-free survival (DFS) rate in 47 HCC cases in which HCC tissues overexpressed α-SMA [48]. Fang et al. also found α-SMA expression in HCC tissues to be negatively associated with DFS [25], and Zou et al. discovered a negative association between CAFs and HCC overall survival (OS). In addition, the proliferation of α-SMA-positive CAFs in HCC tissues was found to be negatively associated with HCC recurrence after liver transplantation [23]. All of the above studies mainly used α-SMA as a biomarker for CAFs, whereas Kim et al. found that FAP expression in HCC tissues did not influence patient survival [24]. These different findings suggest that the identification of more specific biomarkers for CAFs in HCC tissues may improve the ability to predict HCC prognosis and accelerate the clinical application of CAFs.

**Table 2:** CAFs are potential pathological indicators for predicting HCC prognosis

Selection criteria of HCC cases	Source/number/staining of HCC specimens	Quantification of immunohistochemistry		Mean DFS/OS			HCC prognosis	Predictive value	Reference
		Methods	Cut-off value	High-density group	Low-density group				
HCC cases underwent curative radical surgery without neo-adjuvant radiotherapy or chemotherapy	Clinical cases/101 HCC patients/α-SMA	Stromal thickness was measured using the CRi Nuance multispectral imaging system	Mean value of stromal thickness: 238.6 μm	DFS: 3 months	DFS: 12 months		Inverse correlation with DFS	Independent prognostic factor for DFS	Fang et al. [25]
No modality other than LDLT available to treat patients with HCC and end-stage liver disease; no extrahepatic metastasis or macrovascular invasion such as portal vein or hepatic vein infiltration	Clinical cases/22 HCC patients/α-SMA	The percentage of α-SMA expression in the stromal area was calculated	α-SMA expression in stromal area: < 1%, < 10%, > 10%	> 10%	< 10%	> 1%	Inverse correlation with DFS and OS	Independent prognostic factor for DFS	Takamura et al. [23]
				–	–	–			
HCC without a history of preoperative treatment	Clinical cases/314 HCC patients/FAP	Scoring was performed according to staining intensity and the percentage of positive cells	Score (positive: 2, moderate staining in ≥5%, and 3, strong staining in ≥ 5% cells; negative: 0, staining in < 5% cells, and 1, weak staining in ≥5%)	–	–		No association with HCC prognosis	–	Kim et al. [24]

## CAFs and HCC-targeted therapy

Because CAFs are promoted in HCC progression, researchers have attempted to utilize CAF-targeted therapy to inhibit HCC progression. Theoretically, CAF-targeted therapy can be mediated by inhibiting the generation of CAFs, directly depleting CAFs by targeting protein markers, normalizing CAFs toward a quiescent state, inducing their acquisition of a tumor-suppressive phenotype, and inhibiting paracrine cytokines or downstream signaling molecules. However, only a few studies have shown the inhibitory effects of these strategies on HCC progression, mainly achieved by blocking the paracrine effect of CAFs. Among all the paracrine cytokines secreted by HCC-associated fibroblasts that influence HCC progression, IL-6 is the most studied and may constitute a target for CAF-targeted therapy. Indeed, Lu et al. indicated that combined IL-6 and PD-L1 blockade can effectively inhibit HCC growth in vivo, and a group treated with both IL-6 and PD-L1 exhibited smaller tumor volumes and longer survival times than a control group [65]. Although few studies have focused on CAF-targeted therapy, it is a hot research topic in HCC-targeted therapy, and more effort is needed to develop CAF-targeted therapy.

## Conclusions and prospective

The present review systematically summarizes research on the heterogeneous tissue distribution, cellular origin, cellular phenotype, biological function, and potential application of HCC-associated fibroblasts in HCC diagnosis and therapy. CAFs in HCC tissues, which are mainly distributed in the fibrous septum, fibrous capsule, and hepatic blood sinusoids of HCC and sporadic HCC cells, may be derived from HSCs, MSCs, ECs, and HCC cells undergoing EMT. Most CAFs present a myofibroblast phenotype that effectively promotes the in vitro proliferation, migration, invasion, and immune escape of HCC cells and their in vivo tumorigenesis, growth, and metastasis. CAFs are also associated with HCC prognosis and may be ideal targets in HCC-targeted therapy. Recognizing the other possible cellular origins of CAFs and the exact mechanisms by which these cell types are transformed into CAFs will be beneficial for clarifying their differential phenotypes and functions. Using more advanced technologies, such as single-cell sequencing and bioinformatic analysis, to identify more specific biomarkers and novel CAF subtypes may further improve our understanding regarding the heterogeneity of CAFs and accelerate their clinical application in HCC diagnosis and treatment.

## Data Availability

Not applicable.

## Abbreviations

CAFs: Cancer-associated fibroblasts
DCs: Dendritic cells
DFS: Disease-free survival
ECM: Extracellular matrix
ECs: Endothelial cells
EMT: Epithelial-mesenchymal transformation
EndMT: Endothelial to mesenchymal transition
FAP: Fibroblast activation protein
FOXQ1: Forkhead box Q1
FSP-1: Fibroblast-specific protein 1
HCC: Hepatocellular carcinoma;
HSCs: Hepatic stellate cells
HSECs: Hepatic sinusoidal endothelial cells
IF: Immunofluorescence
MDSCs: Myeloid-derived suppressor cells
MSCs: Mesenchymal stem cells
NDRG1: N-myc downstream-regulated gene 1
OS: Overall survival
PDGFRs: Platelet-derived growth factor receptors
PTFs: Peritumor fibroblasts
TME: Tumor microenvironment
VM: Vasculogenic mimicry
α-SMA: α-smooth muscle actin
